# Effector Functions of Natural Killer Cell Subsets in the Control of Hematological Malignancies

**DOI:** 10.3389/fimmu.2015.00567

**Published:** 2015-11-05

**Authors:** Angela Gismondi, Helena Stabile, Paolo Nisti, Angela Santoni

**Affiliations:** ^1^Department of Molecular Medicine, Istituto Pasteur-Fondazione Cenci Bolognetti, Sapienza University of Rome, Rome, Italy; ^2^Eleonora Lorillard Spencer Cenci Foundation, Rome, Italy; ^3^Italian Institute of Technology, Rome, Italy

**Keywords:** NK cell subsets, hematological malignancies, HSCT, NK cell therapy, NK cells

## Abstract

Treatment of hematological malignant disorders has been improved over the last years, but high relapse rate mainly attributable to the presence of minimal residual disease still persists. Therefore, it is of great interest to explore novel therapeutic strategies to obtain long-term remission. Immune effector cells, and especially natural killer (NK) cells, play a crucial role in the control of hematological malignancies. In this regard, the efficiency of allogeneic stem cell transplantation clearly depends on the immune-mediated graft versus leukemia effect without the risk of inducing graft versus host disease. Alloreactive donor NK cells generated following hematopoietic stem cell transplantation ameliorate the outcome of leukemia patients; in addition, *in vivo* transfer of *in vitro* expanded NK cells represents a crucial tool for leukemia treatment. To improve NK cell effector functions against resistant leukemia cells, novel immunotherapeutic strategies are oriented to the identification, isolation, expansion, and administration of particular NK cell subsets endowed with multifunctional anti-tumor potential and tropism toward tumor sites. Moreover, the relationship between the emergence and persistence of distinct NK cell subsets during post-graft reconstitution and the maintenance of a remission state is still rather unclear.

## Introduction

Natural killer (NK) cells belong to innate lymphocytes that play an important role in the early phase of immune defense against microbial infections, and tumor growth and dissemination. They represent a population of highly specialized large granular lymphocytes capable of mediating cytotoxic activity and endowed with the ability to release cytokines and chemokines when properly activated by target cells or pro-inflammatory stimuli (1–3). During microbial infection or tumor growth and invasion, NK cells can be rapidly recruited to and accumulate in the parenchyma of injured organs, contributing to the elimination of infected or transformed cells as well as to the recruitment and activation of other immune cells (4, 5). Thus, NK cells are important effectors in the early phase of innate immune responses and play a crucial role as immune regulators of adaptive immunity (6).

Activation of NK cell functional program results from a delicate balance of signals initiated by a complex receptor system formed by both inhibitory and activating receptors. These receptors are acquired during differentiation, and are oligoclonally distributed on mature NK cells; in most instances, the inhibitory signals override the triggering ones (2).

The inhibitory receptors that mainly bind to MHC class I molecules are grouped into two classes: the killer cell immunoglobulin-like receptor (KIR) family that includes receptors for human leukocyte antigen (HLA)-A, -B, and -C group of alleles, and the C-type lectin receptors, such as CD94/NKG2A that binds to the non-classical HLA class I molecule, HLA-E.

Both the inhibitory receptor families have also an activating counterpart with similar specificity but different ligand affinity, showing inhibitory receptors greater ligand affinity with respect to their activating counterpart. The MHC I activating receptors possess a short intracellular domain and associate with a transducing chain that initiates the activating signaling pathway when engaged by ligands (7–9). The human KIR family consists of 13 genes and 2 pseudogenes, and displays a high degree of diversity that arises from both variability in KIR content and allelic polymorphisms. KIR genes are variably inherited by individuals and expressed by NK cells in an oligoclonal manner.

Among non-MHC I NK cell activating receptors, the best studied is the low-affinity Fc-γ receptor IIIA (CD16) involved in the NK cell-mediated antibody-dependent cellular cytotoxicity (ADCC) (10). Another important activating receptor is NKG2D that binds to self-molecules undergoing up-regulation on stressed, infected, or damaged cells belonging to MIC and ULBP families (11). In addition, NK cell activating receptors also include NKp44, NKp46, and NKp30 Ig-like molecules, collectively termed natural cytotoxicity receptors (NCR), and DNAM-1 (CD226) that cooperatively triggers natural killing (12–14).

Activating and inhibitory receptors are acquired during NK cell differentiation and activation, and are selectively expressed on distinct NK cell subsets. Recognition of MHC I receptors during NK cell development is critical for the acquisition of the functional competence through a process defined as NK cell education or licensing (15, 16). Thus, based on the receptor repertoire and expression levels, phenotypically distinct mature NK cell populations have been identified and suggested to represent specialized subsets mediating different functions and endowed with distinct migratory properties (17).

Natural killer cell differentiation primarily occurs in the bone marrow (BM), although NK cell progenitors can undergo final maturation also in the periphery, and the existence of a thymic pathway of NK cell differentiation has been also described in mice (18–20).

Fully mature, NK cells mainly circulate in the peripheral blood (PB) but they can be also found in several lymphoid and non-lymphoid organs, such as spleen, tonsils, lymph nodes, liver, intestine, lungs, and uterus (1, 21–24). PB NK cells represent about 5–20% of total lymphocytes.

Two major human NK cell subsets, namely, CD56^high^CD16^+/−^ and CD56^low^CD16^high^, can be distinguished in the PB based on the expression levels of the low-affinity Fc-receptor γ IIIA (CD16) and the neural cell adhesion molecule (NCAM, CD56). CD56^high^CD16^+/−^ NK cells primarily secrete immunoregulatory cytokines, whereas the CD56^low^CD16^high^ NK cell subset is the major killer population mediating both natural cytotoxic activity and ADCC. It is still matter of debate whether these subsets are functionally distinct terminally differentiated NK cells or NK cells at a different stage of maturation (17, 25). A sequential relation between these two major NK cell subsets has been recently reported in that it has been shown that CD56^high^ NK cells have longer telomeres than CD56^low^ NK cells and can differentiate into CD56^low^ in humanized mice in the presence of human IL-15, thus suggesting that they represent a more immature stage (26–28).

**Figure 1:**
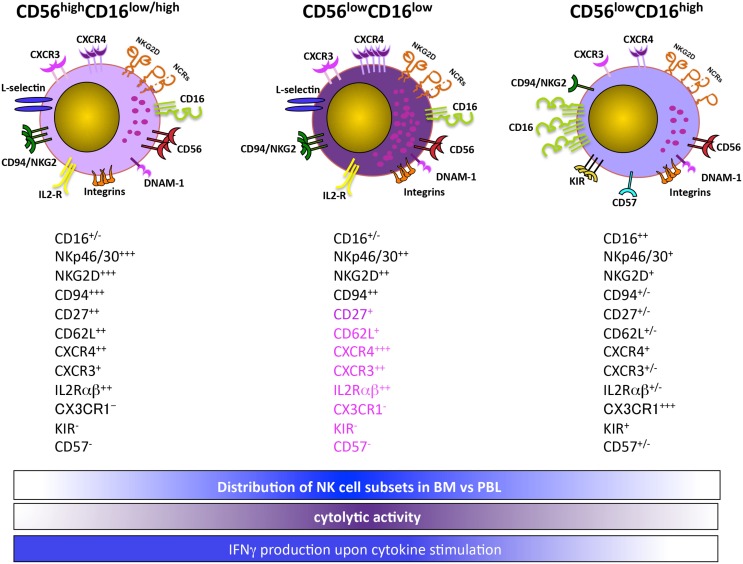
**CD56^low^CD16^low^ NK cells are a functionally and phenotypically distinct NK cell subset**.

Recently, authors identified a distinct CD56^low^ NK cell subset based on CD16 expression levels in the BM and PB of healthy children and acute lymphoblastic leukemia (ALL) pediatric patients (Figure 1). The CD56^low^CD16^low^ NK cells are more prominent in the BM, and in this organ their frequency further increases in ALL children. Both BM and PB CD56^low^CD16^low^ NK cells release IFNγ upon IL-12 plus IL-15 stimulation, and are the major killer population against K562 erythroleukemia cells. However, unlike healthy donors, BM and PB CD56^low^CD16^low^ NK cells from ALL children poorly degranulate in response to K562 target cell stimulation. Interestingly, using PB NK cell subsets from two haploidentical HSC donors as source of effector cells and the leukemic blasts of the corresponding recipients as targets, CD56^low^CD16^low^ NK cells are the unique population capable of killing leukemic blasts (29).

Overall, our findings suggest that CD56^low^CD16^low^ NK cells represent an intermediate state between CD56^high^ and CD56^low^CD16^high^ NK cells. However, the lower levels of CD16 may also imply that CD56^low^CD16^low^ NK cells represent a post-activation stage, as a disintegrin and metalloproteinase-17 (ADAM-17)-dependent CD16 shedding can occur following NK cell activation (30, 31). In this regard, in accordance with the increased number of CD56^low^CD16^low^ NK cells in leukemic children, our preliminary data indicate that ADAM-17 is significantly more abundant in their BM plasma as compared to healthy donors (32).

## NK Cells for Hematological Cancer Therapy: Alloreactive NK Cells

The ability of NK cells to kill leukemic cells in mice without T-cell involvement and prior sensitization was first reported in 1975 (33, 34). Thereafter, growing evidence showed that NK cells preferentially lyse target cells expressing lower or aberrant MHC class I molecules. Based on this evidence, Kärre et al. formulated the missing self hypothesis, arguing that NK cells survey the body for the expression of self-MHC class I molecules and destroy cells on which they are missing (35). Only at the beginning of the 1990s, MHC I inhibitory receptors were discovered in the mouse and humans, and they were shown to deliver negative signals to the NK cells, thus, preventing their cytotoxic activity (2). Accordingly, the use of human NK cell clones revealed that NK cells are unable to lyse autologous normal cells when they express inhibitory receptors for at least one self class I allele. Subsequent findings indicated that NK cells are endowed with alloreactivity and can lyse target cells lacking MHC I molecules (36). This notion has had important clinical implications.

The NK cell therapeutic potential was clearly demonstrated when a potent anti-leukemic effect was observed in patients with acute myeloid leukemia (AML) undergoing mismatched/haploidentical hematopoietic stem cell transplantation (HSCT). This protocol of transplantation relied on the generation of alloreactive NK cells with a KIR repertoire unable to bind to host MHC class I molecules, and was associated with a 65% probability of disease-free survival, decreased incidence of relapse, and no increased incidence of graft versus host disease (GVHD) in T-cell-depleted transplants (37). However, patients with KIR-ligand incompatibility can be still at high risk of GVHD as T-cell alloreactivity may dominate NK cell alloreactivity in minimally T-cell-depleted grafts and in T-cell-repleted transplants (38). The AML-specific effect in unrelated donor transplantation was observed only under particular conditions, including infusion of high doses of stem cells, almost complete depletion of T-cells, no post-grafting immunosuppression, and donors selected for the perfect mismatch at HLA loci (39).

The KIR genes are polymorphic and are organized into two broad haplotypes: group A KIR haplotype, which encodes mainly inhibitory receptors (KIR2DL2/3, KIR3DL2/3, KIR2DL4, KIR3DL1) and one activating receptor (KIR2DS4), and group B KIR haplotype, which encodes both inhibitory (KIR2DL2/3, KIR3DL2/3, KIR2DL5B/A, KIR2DL4, KIR3DL1) and several activating KIR (KIR2DS1, KIR2DS2, KIR2DS3, KIR2DS5, KIR3DS1) (Table 1) (40, 41).colorno6rgb0.850980392156863, 0.850980392156863, 0.850980392156863

**Table 1:** **Killer immunoglobulin-like (KIR) haplotypes**.

	HLA class I ligand	Inhibitory	Activating
**A. Haplotype**
3DL2	HLA-A(A*3, A*11)	+	
2DS4	HLA-C(C*5, C*16, A*11)		+
3DL1	Bw4	+	
3DS1	Bw4?		+
2DL4	HLA-G	+	
DP1		−	
DL1	HLA-C(C2)	+	
DP1		−	
DL2	HLA-C(Cl/c2, few HLA-B)	+	
DL3	HLA-C(Cl/c2, few HLA-B)	+	
DL3	N.D	+	
**B. Haplotype**
3DL2	HLA-A(A*3, A*11)	+	
2DS4	HLA-C(C*5, C*16, A*11)		+
2DS1	HLA-C(C2)		+
2DS3/5	N.D		+
2DL5A	N.D	+	
3DL1	Bw4	+	
3DS1	Bw4?		+
2DL4	HLA-G		+
DP1		−	
DL1	HLA-C(C2)	+	
DP1		−	
DS3/5	N.D		+
DL5B	N.D	+	
DL2	HLA-C(Cl/c2, few HLA-B)	+	
DL3	HLA-C(Cl/c2, few HLA-B)	+	
DS2	A11?		+
DL3	N.D	+	

*Gray and white box contain telomeric and centrometric KIRs respectively; C1/c2 denotes a strong reaction with C1 and weaker cross-reaction with C2; ? denotes uncertainty*.

Although the initial findings outlined the importance of inhibitory KIRs on the outcome of HSCT, several later studies also focused attention on the influence of activating KIRs. Thus, AML patients transplanted with HSC from donor with alloreactive NK cells carrying KIR2DS1 displayed a lower rate of leukemia relapse as compared to those carrying KIR3DS1 who had no effect on leukemic disease but showed a reduced risk of infection-related mortality (42). Similarly, Mancusi et al. reported that transplantation from donors with KIR2DS1 and/or KIR3DS1 resulted in reduced non-relapse mortality and improved survival (43).

The KIR protective effects were dependent on high levels of HLAC ligands and was restricted to donors with HLA-C1/C1 or HLA C1/C2, whereas it was lost if the donors were HLA C2/C2. This is probably due to the high levels of activating HLA C2 ligands that could induce NK cell tolerance, thus impairing their anti-leukemic effector functions (42).

Variation of the KIR gene family and the impact of KIR-ligand mismatch on the outcome of HSCT have been also addressed. Kröger et al. analyzed the KIR haplotypes in leukemic patients who received T-cell-depleted unrelated HSCT. They observed that only patients that have received transplants from donors carrying group A haplotype or a small number of activating KIR genes, exhibit reduced relapse and increased disease-free survival. This effect was observed only for AML/myelodysplastic syndrome and to a lesser extent for chronic myeloid leukemia, whereas no effect was evidentiated for ALL (44). The influence of donor and recipient KIR genotype on the outcome of HSCT between HLA-matched siblings was also reported by McQueen et al. Transplants were divided in four groups according to the combination of A and B KIR genotype in the donor and recipient. Better survival was found to be associated with the donor lacking and the recipient having group B KIR haplotype. When haplotype B was present in the donor and absent in the recipient, increased relapse and acute GVHD was observed only if recipient and donor were homogyzous for HLAC1 KIR ligand and lacked HLAC2 ligand.

These findings could be attributable to the presence of activating KIRs in the graft, with a preferential promotion of GVHD but not GVL response, as the activating KIRs on grafted NK or T cells might cause host alloaggression and impaired reconstitution of a responsive immune system (45). Conversely, a multicenter analysis demonstrated a significant and substantial survival benefit for AML patients receiving grafts from unrelated donors having 1 or 2 KIR B haplotypes, thus providing evidence that donors with KIR B haplotype should be used preferentially in HLA-matched or HLA-mismatched unrelated donor transplantation (46). In addition, it is not the presence of the activating B haplotype by itself, but rather the presence of three particular donor genes (2DL5A, 2DS1, and 3DS1) within the B haplotype that is associated with reduced relapse (47). Moreover, AML patients transplanted with HSC from donor with alloreactive NK cells carrying KIR2DS1 and/or KIR3DS1 also display a reduced mortality related to infections, and thus a better event-free survival (42, 43).

Although many reports demonstrate a therapeutic role for alloreactive NK cells in AML, evidence are also available on the importance of KIR-HLA matching. In this regard, Farag et al. analyzed the outcome of 1571 unrelated donor–recipient transplanted patients with myeloid malignancies by comparing donor–recipient pairs, such as HLA-A, -B, -C, and -DRB1 matched, KIR-ligand-mismatched, and HLA-B and/or-C-mismatched but KIR-ligand-matched, and reported that treatment-related mortality, treatment failure, and overall mortality were lowest only after matched transplantation (48).

No clear benefit has been observed for haploidentical HSCT in adult ALL patients with respect to AML patients (37, 44). However, successful results have been obtained in children with ALL transplanted with haploidentical T cell depleted HSC (49). A possible explanation for the distinct role of alloreactive NK cells in pediatric versus adult ALL transplanted patients stems from a recent report demostrating a differential activating ligand repertoire on the leukemic cells. Pediatric ALL blasts exhibited higher expression levels of both the DNAM-1 ligand Nec-2, and the NKG2D ligands ULBP-1 and ULBP-3, as compared to adult ALL blasts (50).

## NK Cell Reconstitution after HSCT and Adoptive Infusion of NK Cells

After HSCT, the first class of lymphocytes which reconstitute in the PB are NK cells that precede T cell reconstitution. The earliest reconstituted NK cells exhibit a CD56^high^ phenotype and express high levels of NKG2A/CD94 and lower levels of inhibitory KIR (51, 52). The analysis of the functional ability of NK cells reconstituted after adult unrelated donor or umbilical cord blood grafting reveal that NK cells from T cell-depleted transplant recipients without immunosuppression, display poor degranulating ability, whereas degranulation is normal or increased in patients undergoing T-cell-repleted transplants and receiving immunosuppression. Based on this observation, a role for T cells in NK cell education and KIR acquisition has been suggested (53). However, another study on the comparison of NK cell reconstitution in T cell-repleted and T cell-depleted HLA-matched sibling, HSCT, indicates that functional recovery of both uneducated and educated NK cells is similar in T cell-depleted and T cell-repleted settings. Moreover, NKG2A^+^ donor NK cells are predominant early after transplantation before expression of KIR, whereas the NKG2A^−^ NK cells expressing KIR for non-self ligand, remain tolerant in both settings, suggesting that NK cell subsets expressing inhibitory receptors for non-self HLA class I molecules remain hyporesponsive after HLA-matched HSCT (54).

Overall, the analysis of the NK cell repertoire and functional ability at different times after transplantation reveal a marked hyporesponsiveness of NK cells early after transplantation (55).

Moreover, cytokine producing and degranulating abilities are not co-expressed in reconstituting NK cells after allo-HSCT, as target cell-induced IFNγ production is markedly diminished in all transplant settings, whereas the cytotoxic activity is impaired only in T cell-depleted HSCT. The decreased ability to produce IFNγ is rapidly reverted by exposure to low dose of IL-15 suggesting a potential therapeutic role for this cytokine by enhancing NK cell protective ability against infection and relapse (56).

In accordance with these findings, our preliminary results on the CD56^low^CD16^low^ NK cells indicate that like CD56^high^ NK cells this subset is present in the PB and BM already at 1 month post-T cell-depleted HSCT, and their number is increased with respect to healthy individuals. However, unlike healthy donors, no differences in CD56^low^CD16^low^ NK cell distribution between the two tissue compartments during the first 6 months after HSCT are observed. In transplanted patients, CD56^low^CD16^low^ NK cells produce higher levels of IFNγ after IL-12 plus IL-15 stimulation, but they are still the only NK cell degranulating subset when challenged with K562 cells, although the extent of degranulation is lower than that of healthy controls (Stabile et al. unpublished observations).

Adoptive transfer of NK cells have been also considered a promising therapeutic option in the treatment of hematological malignancies, especially in T-cell-depleted haplo-SCT setting because their potent GVL effect (55, 56).

The early clinical trials based on adoptive cell transfer, utilized lymphokine-activated killer (LAK) cells generated from autologous PBMCs cultured *in vitro* with high doses IL-2 for 3–7 days in order to induce anti-tumor killer cells that mainly consisted of NK cells (57–59). Systemic high doses of IL-2 were also administered in order to activate the autologous NK cells *in vivo*; however, in this case, severe toxicity occurred due to capillary leak syndrome induced by IL-2 (59, 60). Subcutaneous administration of low doses of IL-2 alone or in combination with LAK cells gave encouraging results only in patients with melanoma and renal carcinoma (61, 62). Although these approaches augmented *in vivo* activity of NK cells, no consistent efficacy of autologous NK-cell therapy could be detected in patients with other cancer histotypes, including hematological malignancies (62).

The failure of this kind of immunotherapy has been attributed to downregulation of NK cell activity by KIR engagement by self-MHC (37), competition with recipient’s lymphocytes for cytokines and space, chronic immunosuppression induced by tumor and/or expansion of Treg cells by IL-2 (63, 64). However, by analyzing cancer patients (metastatic melanoma, renal cell carcinoma, refractory Hodgkin’s disease, and refractory AML) subjected to adoptive transfer of human NK cells from haploidentical-related donors, Miller and collaborators demonstrated that transferred NK cells can be expanded *in vivo* and that expansion is dependent on the more intense cyclophosphamide/fludarabine chemotherapy regimen that induces lymphopenia and high endogenous concentrations of IL-15, which are not observed when lower doses of chemotherapy are administered. More importantly, 5 of 19 poor-prognosis AML patients achieved complete remission after haploidentical NK cell therapy, with a significant higher complete remission rate when KIR-ligand mismatched donors were used (65). In accordance with the presence of high concentrations of IL-15 mature NK cells transferred in high-risk AML patients undergoing haploHSCT, were found to proliferate *in vivo* during the early days after haplo HSCT even in the absence of exogenous IL-2 administration, and this resulted in relative low patient relapse rate (66). Moreover, IL-15 together with IL-12 and IL-18 was reported to increase the expression of high affinity IL-2 receptor that was associated with increased NK cell survival, proliferation, and effector function, thus leading to propose immunotherapeutic strategies based on short cytokine preactivation of NK cell before adoptive transfer and followed by low doses of IL-2 therapy (67, 68).

The existence of NK cell subsets with distinct phenotype, functional ability, and adhesion and chemotactic properties that drive their tropism to different tissue compartments, strongly suggests that NK cell-based therapies still require a better identification of NK cell subsets endowed with optimal anti-tumor potential and tropism to tumor sites, to achieve optimal clinical benefit.

## Conclusion

Authors suppose that the identification and characterization of multifunctional NK cell subsets, which can be rapidly mobilized in the PB and with strong ability to migrate to tumor sites, would provide new insights on the role played by NK cells under pathological conditions and, more importantly, would allow the design of new approaches of adoptive immunotherapy to treat patients with NK cell-susceptible hematological malignancies. Further studies would also clarify the relationship between emergence and persistence of distinct NK cell subsets during post-graft reconstitution and the maintenance of a state of remission.

## Conflict of Interest Statement

The authors declare that the research was conducted in the absence of any commercial or financial relationships that could be construed as a potential conflict of interest.

## References

[1] Trinchieri G Biology of natural killer cells. Adv Immunol (1989) 47:187–376. 10.1016/S0065-2776(08)60664-1 2683611 PMC7131425

[2] Lanier LL. NK cell recognition. Annu Rev Immunol (2005) 23:225–74. 10.1146/annurev.immunol.23.021704.115526 15771571

[3] Vivier E Tomasello E Baratin M Walzer T Ugolini S. Functions of natural killer cells. Nat Immunol (2008) 9:503–10. 10.1038/ni1582 18425107

[4] Biron CA Activation and function of natural killer cell responses during viral infection. Curr Opin Immunol (1997) 9:24–34. 10.1016/S0952-7915(97)80155-0 9039782

[5] Fogler WE Volker K McCormick KL Watanabe M Ortaldo JR Wiltrout RH. NK cell infiltration into lung, liver, and subcutaneous B16 melanoma is mediated by VCAM-1/VLA-4 interaction. J Immunol (1996) 156:4707–14. 8648116

[6] Moretta A. Natural killer cells and dendritic cells: rendezvous in abused tissues. Nat Rev Immunol (2002) 2:957–64. 10.1038/nri956 12461568

[7] Valés-Gómez M Reyburn HT Mandelboim M Strominger JL. Kinetics of interaction of HLA-C ligands with natural killer cell inhibitory receptors. Immunity (1998) 9:337–44. 10.1016/S1074-7613(00)80616-0 9768753

[8] Biassoni R Cantoni C Falco M Verdiani S Bottino C Vitale M The human leukocyte antigen (HLA)-C-specific “activatory” or “inhibitory” natural killer cell receptors display highly homologous extracellular domains but differ in their transmembrane and intracytoplasmic portions. J Exp Med (1996) 183:645–50. 10.1084/jem.183.2.645 8627176 PMC2192451

[9] Campbell KS Cella M Carretero M López-Botet M Colonna M. Signaling through human killer cell activating receptors triggers tyrosine phosphorylation of an associated protein complex. Eur J Immunol (1998) 28:599–609. 10.1002/(SICI)1521-4141(199802)28:02<599::AID-IMMU599>3.3.CO;2-6 9521070

[10] Trinchieri G Valiante N. Receptors for the Fc fragment of IgG on natural killer cells. Nat Immunol (1993) 12:218–34. 8257828

[11] Moretta A Bottino C Vitale M Pende D Cantoni C Mingari MC Activating receptors and coreceptors involved in human natural killer cell-mediated cytolysis. Annu Rev Immunol (2001) 19:197–223. 10.1146/annurev.immunol.19.1.197 11244035

[12] Gilfillan S Chan CJ Cella M Haynes NM Rapaport AS Boles KS DNAM-1 promotes activation of cytotoxic lymphocytes by nonprofessional antigen-presenting cells and tumors. J Exp Med (2008) 205:2965–73. 10.1084/jem.20081752 19029380 PMC2605240

[13] Bryceson YT March ME Ljunggren HG Long EO. Activation, coactivation, and costimulation of resting human natural killer cells. Immunol Rev (2006) 214:73–91. 10.1111/j.1600-065X.2006.00457.x 17100877 PMC3845883

[14] Gasser S Orsulic S Brown EJ Raulet DH. The DNA damage pathway regulates innate immune system ligands of the NKG2D receptor. Nature (2005) 436:1186–90. 10.1038/nature03884 15995699 PMC1352168

[15] Brodin P Lakshmikanth T Johansson S Kärre K Höglund P. The strength of inhibitory input during education quantitatively tunes the functional responsiveness of individual natural killer cells. Blood (2009) 113:2434–41. 10.1182/blood-2008-05-156836 18974374

[16] Joncker NT Fernandez NC Treiner E Vivier E Raulet DH. NK cell responsiveness is tuned commensurate with the number of inhibitory receptors for self-MHC class I: the rheostat model. J Immunol (2009) 182:4572–80. 10.4049/jimmunol.0803900 19342631 PMC2938179

[17] Cooper MA Fehniger TA Turner SC Chen KS Ghaheri BA Ghayur T Human natural killer cells: a unique innate immunoregulatory role for the CD56(bright) subset. Blood (2001) 97:3146–51. 10.1182/blood.V97.10.3146 11342442

[18] Freud AG Caligiuri MA. Human natural killer cell development. Immunol Rev (2006) 214:56–72. 10.1111/j.1600-065X.2006.00451.x 17100876

[19] Di Santo JP Vosshenrich CAJ. Bone marrow versus thymic pathways of natural killer cell development. Immunol Rev (2006) 214:35–46. 10.1111/j.1600-065X.2006.00461.x 17100874

[20] Di Santo JP. Natural killer cell developmental pathways: a question of balance. Annu Rev Immunol (2006) 24:257–86. 10.1146/annurev.immunol.24.021605.090700 16551250

[21] Fehniger TA Cooper MA Nuovo GJ Cella M Facchetti F Colonna M CD56^bright^ natural killer cells are present in human lymph nodes and are activated by T cell-derived IL-2: a potential new link between adaptive and innate immunity. Blood (2003) 101:3052–7. 10.1182/blood-2002-09-2876 12480696

[22] King A Burrows T Loke YW. Human uterine natural killer cells. Nat Immun (1996-1997) 15:41–52. 9032767

[23] Whitelaw PF Croy BA. Granulated lymphocytes of pregnancy. Placenta (1996) 17:533–43. 10.1016/S0143-4004(96)80070-1 8916201

[24] Santoni A Carlinoc C Gismondi A. Uterine NK cell development, migration and function. Reprod Biomed Online (2008) 16:202–10. 10.1016/S1472-6483(10)60575-5 18284874

[25] Perussia B Loza MJ Linear “2-0-1” lymphocyte development: hypotheses on cellular bases for immunity. Trends Immunol (2003) 24:235–41. 10.1016/S1471-4906(03)00080-2 12738416

[26] Ouyang Q Baerlocher G Vulto I Lansdorp PM. Telomere length in human natural killer cell subsets. Ann N Y Acad Sci (2007) 1106:240–52. 10.1196/annals.1392.001 17303822

[27] Romagnani C Juelke K Falco M Morandi B D’Agostino A Costa R CD56brightCD16- killer Ig-like receptor-NK cells display longer telomeres and acquire features of CD56dim NK cells upon activation. J Immunol (2007) 178:4947–55. 10.4049/jimmunol.178.8.4947 17404276

[28] Huntington ND Legrand N Alves NL Jaron B Weijer K Plet A IL-15 trans-presentation promotes human NK cell development and differentiation in vivo. J Exp Med (2009) 206:25–34. 10.1084/jem.20082013 19103877 PMC2626663

[29] Stabile H Nisti P Morrone S Pagliara D Bertaina A Locatelli F Multifunctional human CD56low CD16low natural killer cells are the prominent subset in bone marrow of both pediatric healthy donors and leukemic patients. Haematologica (2015) 100:489–98. 10.3324/haematol.2014.116053 25596273 PMC4380722

[30] Romee R Foley B Lenvik T Wang Y Zhang B Ankarlo D NK cell CD16 surface expression and function is regulated by a disintegrin and metalloprotease-17 (ADAM17). Blood (2013) 121:3599–608. 10.1182/blood-2012-04-425397 23487023 PMC3643761

[31] Lajoie L Congy-Jolivet N Bolzec A Gouilleux-Gruart V Sicard E Sung HC ADAM17-mediated shedding of FcγRIIIA on human NK cells: identification of the cleavage site and relationship with activation. J Immunol (2014) 192:741–51. 10.4049/jimmunol.1301024 24337742

[32] Stabile H Nisti P Pagliara D Locatelli F Santoni A Gismondi A Response to comment on multifunctional human CD56^low^CD16^low^ NK cells are the prominent subset in bone marrow of both pediatric healthy donors and leukemic patients. Haematologica (2015) 100(8):e332–3. 10.3324/haematol.2014.116053 26314084 PMC5004438

[33] Kiessling R Klein E Wigzell H “Natural” killer cells in the mouse. I. Cytotoxic cells with specificity for mouse Moloney leukemia cells. Specificity and distribution according to genotype. Eur J Immunol (1975) 5:112–21. 10.1002/eji.1830050208 1234049

[34] Herberman RB Nunn ME Lavrin DH. Natural cytotoxic reactivity of mouse lymphoid cells against syngeneic acid allogeneic tumors. I. Distribution of reactivity and specificity. Int J Cancer (1975) 16:216–39. 10.1002/ijc.2910160204 50294

[35] Ljunggren HG Kärre K. In search of the ‘missing self’: MHC molecules and NK cell recognition. Immunol Today (1990) 11:237–44. 10.1016/0167-5699(90)90097-S 2201309

[36] Moretta L Ciccone E Moretta A Höglund P Ohlén C Kärre K. Allorecognition by NK cells: nonself or no self? Immunol Today (1992) 8:300–6. 10.1016/0167-5699(92)90042-6 1380815

[37] Ruggeri L Capanni M Urbani E Perruccio K Shlomchik WD Tosti A Effectiveness of donor natural killer cell alloreactivity in mismatched hematopoietic transplants. Science (2002) 295:2097–100. 10.1126/science.1068440 11896281

[38] Lowe EJ Turner V Handgretinger R Horwitz EM Benaim E Hale GA T-cell alloreactivity dominates natural killer cell alloreactivity in minimally T-cell-depleted HLA-non-identical paediatric bone marrow transplantation. Br J Haematol (2003) 123:323–6. 10.1046/j.1365-2141.2003.04604.x 14531915

[39] Davies SM Ruggieri L DeFor T Wagner JE Weisdorf DJ Miller JS Evaluation of KIR ligand incompatibility in mismatched unrelated donor hematopoietic transplants. Killer immunoglobulin-like receptor. Blood (2002) 100:3825–7. 10.1182/blood-2002-04-1197 12393440

[40] Guethlein LA Norman PJ Hilton HH Parham P. Co-evolution of MHC class I and variable NK cell receptors in placental mammals. Immunol Rev (2015) 267:259–82. 10.1111/imr.12326 26284483 PMC4587382

[41] Thielens A Vivier E Romagné F. NK cell MHC class I specific receptors (KIR): from biology to clinical intervention. Curr Opin Immunol (2012) 24:239–45. 10.1016/j.coi.2012.01.001 22264929

[42] Venstrom JM Pittari G Gooley TA Chewning JH Spellman S Haagenson M HLA-C-dependent prevention of leukemia relapse by donor activating KIR2DS1. N Engl J Med (2012) 367:805–16. 10.1056/NEJMoa1200503 22931314 PMC3767478

[43] Mancusi A Ruggeri L Urbani E Pierini A Massei MS Carotti A Haploidentical hematopoietic transplantation from KIR ligand-mismatched donors with activating KIRs reduces non-relapse mortality. Blood (2015) 125:3173–82. 10.1182/blood-2014-09-599993 25769621

[44] Kröger N Binder T Zabelina T Wolschke C Schieder H Renges H Low number of donor activating killer immunoglobulin-like receptors (KIR) genes but not KIR-ligand mismatch prevents relapse and improves disease-free survival in leukemia patients after in vivo T-cell depleted unrelated stem cell transplantation. Transplantation (2006) 82:1024–30. 10.1097/01.tp.0000235859.24513.43 17060849

[45] McQueen KL Dorighi KM Guethlein LA Wong R Sanjanwala B Parham P. Donor-recipient combinations of group A and B KIR haplotypes and HLA class I ligand affect the outcome of HLA-matched, sibling donor hematopoietic cell transplantation. Hum Immunol (2007) 68:309–23. 10.1016/j.humimm.2007.01.019 17462498 PMC1937576

[46] Cooley S Trachtenberg E Bergemann TL Saeteurn K Klein J Le CT Donors with group B KIR haplotypes improve relapse-free survival after unrelated hematopoietic cell transplantation for acute myelogenous leukemia. Blood (2009) 113:726–32. 10.1182/blood-2008-07-171926 18945962 PMC2628378

[47] Stringaris K Adams S Uribe M Eniafe R Wu CO Savani BN Donor KIR Genes 2DL5A, 2DS1 and 3DS1 are associated with a reduced rate of leukemia relapse after HLA-identical sibling stem cell transplantation for acute myeloid leukemia but not other hematologic malignancies. Biol Blood Marrow Transplant (2010) 16:1257–64. 10.1016/j.bbmt.2010.03.004 20302958 PMC3801172

[48] Farag SS Bacigalupo A Eapen M Hurley C Dupont B Caligiuri MA The effect of KIR ligand incompatibility on the outcome of unrelated donor transplantation: a report from the center for international blood and marrow transplant research, the European blood and marrow transplant registry, and the Dutch registry. KIR Study Group, Center for International Blood and Marrow Transplantation Research. Biol Blood Marrow Transplant (2006) 12:876–84. 10.1016/j.bbmt.2006.05.007 16864058

[49] Oevermann L Michaelis SU Mezger M Lang P Toporski J Bertaina A KIR B haplotype donors confer a reduced risk for relapse after haploidentical transplantation in children with ALL. Blood (2014) 124:2744–7. 10.1182/blood-2014-03-565069 25115891 PMC4208288

[50] Torelli GF Peragine N Raponi S Pagliara D De Propris MS Vitale A Recognition of adult and pediatric acute lymphoblastic leukemia blasts by natural killer cells. Haematologica (2014) 99:1248–54. 10.3324/haematol.2013.101931 24658822 PMC4077088

[51] Nguyen S Dhedin N Vernant JP Kuentz M Al Jijakli A Rouas-Freiss N NK-cell reconstitution after haploidentical hematopoietic stem-cell transplantations: immaturity of NK cells and inhibitory effect of NKG2A override GvL effect. Blood (2005) 105:4135–42. 10.1182/blood-2004-10-4113 15687235

[52] Vago L Forno B Sormani MP Crocchiolo R Zino E Di Terlizzi S Temporal, quantitative, and functional characteristics of single-KIR-positive alloreactive natural killer cell recovery account for impaired graft-versus-leukemia activity after haploidentical hematopoietic stem cell transplantation. Blood (2008) 112:3488–99. 10.1182/blood-2007-07-103325 18645039

[53] Foley B Cooley S Verneris MR Curtsinger J Luo X Waller EK NK cell education after allogeneic transplantation: dissociation between recovery of cytokine-producing and cytotoxic functions. Blood (2011) 118:2784–92. 10.1182/blood-2011-04-347070 21757615 PMC3172795

[54] Björklund AT Schaffer M Fauriat C Ringdén O Remberger M Hammarstedt C NK cells expressing inhibitory KIR for non-self-ligands remain tolerant in HLA-matched sibling stem cell transplantation. Blood (2010) 115:2686–94. 10.1182/blood-2009-07-229740 20097883 PMC2852367

[55] Curti A Ruggeri L D’Addio A Bontadini A Dan E Motta MR Successful transfer of alloreactive haploidentical KIR ligand-mismatched natural killer cells after infusion in elderly high risk acute myeloid leukemia patients. Blood (2011) 118:3273–9. 10.1182/blood-2011-01-329508 21791425

[56] Stern M Passweg JR Meyer-Monard S Esser R Tonn T Soerensen J Pre-emptive immunotherapy with purified natural killer cells after haploidentical SCT: a prospective phase II study in two centers. Bone Marrow Transplant (2013) 48:433–8. 10.1038/bmt.2012.162 22941380

[57] Grimm EA Mazumder A Zhang HZ Rosenberg SA. Lymphokine-activated killer cell phenomenon. Lysis of natural killer-resistant fresh solid tumor cells by interleukin 2-activated autologous human peripheral blood lymphocytes. J Exp Med (1982) 155:1823–41. 10.1084/jem.155.6.1823 6176669 PMC2186695

[58] Rosenberg SA Lotze MT Muul LM Leitman S Chang AE Ettinghausen SE Observations on the systemic administration of autologous lymphokine-activated killer cells and recombinant interleukin-2 to patients with metastatic cancer. N Engl J Med (1985) 313:1485–92. 10.1056/NEJM198512053132327 3903508

[59] Rosenberg SA Lotze MT Muul LM Chang AE Avis FP Leitman S A progress report on the treatment of 157 patients with advanced cancer using lymphokine-activated killer cells and interleukin-2 or high-dose interleukin-2 alone. N Engl J Med (1987) 316:889–97. 10.1056/NEJM198704093161501 3493432

[60] Phillips JH Gemlo BT Myers WW Rayner AA Lanier LL. In vivo and in vitro activation of natural killer cells in advanced cancer patients undergoing combined recombinant interleukin-2 and LAK cell therapy. J Clin Oncol (1987) 5:1933–41. 3500280 10.1200/JCO.1987.5.12.1933

[61] Rosenberg SA Lotze MT Yang JC Topalian SL Chang AE Schwartzentruber DJ Prospective randomized trial of high-dose interleukin-2 alone or in conjunction with lymphokine-activated killer cells for the treatment of patients with advanced cancer. J Natl Cancer Inst (1993) 85:622–32. 10.1093/jnci/85.8.622 8468720

[62] Burns LJ Weisdorf DJ DeFor TE Vesole DH Repka TL Blazar BR IL-2-based immunotherapy after autologous transplantation for lymphoma and breast cancer induces immune activation and cytokine release: a phase I/II trial. Bone Marrow Transplant (2003) 32:177–86. 10.1038/sj.bmt.1704086 12838283

[63] Lundqvist A Yokoyama H Smith A Berg M Childs R. Bortezomib treatment and regulatory T-cell depletion enhance the antitumor effects of adoptively infused NK cells. Blood (2009) 113:6120–7. 10.1182/blood-2008-11-190421 19202127 PMC2699233

[64] Zorn E Nelson EA Mohseni M Porcheray F Kim H Litsa D IL-2 regulates FOXP3 expression in human CD4+CD25+ regulatory T cells through a STAT-dependent mechanism and induces the expansion of these cells in vivo. Blood (2006) 108:1571–9. 10.1182/blood-2006-02-004747 16645171 PMC1895505

[65] Miller JS Soignier Y Panoskaltsis-Mortari A McNearney SA Yun GH Fautsch SK Successful adoptive transfer and in vivo expansion of human haploidentical NK cells in patients with cancer. Blood (2005) 105:3051–7. 10.1182/blood-2004-07-2974 15632206

[66] Killig M Friedrichs B Meisig J Gentilini C Blüthgen N Loddenkemper C Tracking in vivo dynamics of NK cells transferred in patients undergoing stem cell transplantation. Eur J Immunol (2014) 44:2822–34. 10.1002/eji.201444586 24895051

[67] Cooper MA Bush JE Fehniger TA VanDeusen JB Waite RE Liu Y In vivo evidence for a dependence on interleukin 15 for survival of natural killer cells. Blood (2002) 100:3633–8. 10.1182/blood-2001-12-0293 12393617

[68] Leong JW Chase JM Romee R Schneider SE Sullivan RP Cooper MA Preactivation with IL-12, IL-15, and IL-18 induces CD25 and a functional high-affinity IL-2 receptor on human cytokine-induced memory-like natural killer cells. Biol Blood Marrow Transplant (2014) 20:463–73. 10.1016/j.bbmt.2014.01.006 24434782 PMC3959288

